# The use of TDM in pregnant HIV-positive women: a retrospective cross-sectional review of five years practice in two large hospitals in Manchester

**DOI:** 10.7448/IAS.17.4.19695

**Published:** 2014-11-02

**Authors:** Thomas Whitfield, Amabel Dessain, Kelly Taylor, Orla McQuillan, Evelyn Looi, Margaret Kingston, Katherine Ajdukiewicz

**Affiliations:** 1 Infectious Diseases, North Manchester General Hospital, University of London, Manchester, UK; 2 Manchester Centre for Sexual Health, Genitourinary Medicine, Manchester, UK; 3 Paediatrics, North Manchester General Hospital, Manchester, UK; 4 Infectious Diseases, North Manchester General Hospital, Manchester, UK

## Abstract

**Introduction:**

Despite plasma levels of certain HIV drugs decreasing in the third trimester of pregnancy there is no definitive evidence that therapeutic drug monitoring (TDM) improves HIV control and prevents mother-to-child transmission (MTCT). Indeed “one-off” TDM measurements are thought to poorly correlate with overall drug exposure [1]. We aim to describe baseline demographic and clinical characteristics of pregnant women with HIV, and to compare their HIV control, management during pregnancy and neonatal outcomes with respect to whether TDM was performed.

**Materials and Methods:**

Retrospective cross-sectional case note analysis was performed on pregnant women with HIV who attended North Manchester General Hospital and Manchester Royal Infirmary from 1st January 2008 to 28th May 2013.

**Results:**

A total of 171 pregnancies were included; 39% (n=66) had TDM. The majority of patients were of African origin (85%) and age range was 16–42 years (median 32 years). TDM was found to be associated with a history of poor adherence to therapy (TDM 23%, vs no TDM 10%, p=0.017), although baseline viral load (VL) and CD4 counts were comparable between TDM and non-TDM groups (p=0.4756 and 0.9492, respectively). TDM was also associated with protease inhibitors (PI) (TDM 94% vs no TDM 77%, p= 0.004). Within the PI group, TDM was more strongly associated with atazanavir use than other PI's (55%, p=0.023). TDM was not associated with any other demographic variable or with either of the two hospital sites (p=0.427). TDM was associated with medication alterations during pregnancy (TDM 67% vs no TDM 13%, p=0.052), but was not associated with any difference in outcomes with similar proportions of newly detectable VL during pregnancy (TDM 12% vs no TDM 7%, p=0.220) and VL detectable at birth (TDM 14% vs no TDM 9%, p= 0.293). There were no instances of MTCT.

**Conclusions:**

TDM was associated with PI use and a history of poor adherence at baseline. TDM was not associated with improved HIV control during pregnancy and there was no MTCT. TDM was not shown to have any additional benefit in pregnancy and its routine use is not recommended to improve HIV control or reduce MTCT.
